# Assessing the Impact of Burnout on Nurse Safety Behaviors and Patient Safety Competence: A Latent Profile Analysis Study

**DOI:** 10.1155/jonm/3793927

**Published:** 2025-03-27

**Authors:** Fengyan Ma, Yajing Zhu, Lu Liu, Helin Chen, Yan Liu, Fan Zhang

**Affiliations:** Department of Thoracic Surgery, National Cancer Center, National Clinical Research Center for Cancer/Cancer Hospital, Chinese Academy of Medical Sciences, Peking Union Medical College, Beijing, China

**Keywords:** burnout, cancer hospitals, latent profile, nurses, nurse safety behaviors, patient safety competency

## Abstract

**Aim:** This study examines the association between burnout, nurse safety behaviors, and patient safety competency among nurses working in cancer hospitals using person-centered and variable-centered methodologies.

**Background:** Burnout is prevalent among nurses worldwide, with cancer hospital nurses exhibiting high levels of burnout. Burnout correlates with a higher incidence of adverse events and diminished patient safety. Nurse safety behaviors and patient safety competency play protective roles in ensuring patient safety.

**Methods:** This study used a cross-sectional online survey and included 2092 eligible nurses, with 95.0% being female. We invited nurses from cancer hospitals in 12 provinces in China to complete an online survey from April to June 2023. Through the online Questionnaire Star platform, invited nurses provided demographic information and completed the Maslach Burnout Inventory, the Nurse Safety Behaviors Scale, and the Patient Safety Competency Scale. Latent profile analysis was used to identify heterogeneous characteristics of nurse burnout.

**Results:** From a person-centered perspective, nurse burnout was categorized into three latent profiles: “high achievement stable type” (70.3%), “high-efficiency contradictory type” (6.6%), and “high-pressure adaptive type” (23.1%). From a variable-centered perspective, patient safety competency partially mediated the relationship between burnout profiles and nurse safety behaviors.

**Conclusion:** This study identified three heterogeneous latent profiles of burnout among cancer hospital nurses and highlighted the significant impact of excessive working hours and lack of safety training on burnout across different job titles and income levels. Additionally, it verified the mediation effect of patient safety competency between burnout profiles and nurse safety behaviors. Future treatments should focus on high-risk populations by offering improved safety training and suitable work schedules to reduce burnout. Furthermore, personalized measures to enhance nurses' safety competencies should be adopted to improve burnout and safety behaviors. This study integrates person-centered and variable-centered methods, offering new insights and underscoring the critical role of safety in mitigating burnout.

## 1. Introduction

Health problems are increasingly becoming a major public health concern due to the rapid socioeconomic growth worldwide [1] . Given their vital role in safeguarding public health, government agencies and society must place greater emphasis on the occupational health of nurses. Burnout is a serious problem that affects nurses worldwide.

The psychological illness known as burnout is caused by extended exposure to work-related stress without adequate management. It poses a serious risk to the occupational health of nurses because it can result in emotional exhaustion, depersonalization, and a lack of personal accomplishment [2–4]. China now suffers from an imbalance in the allocation of nursing personnel throughout provinces and cities, which increases workloads, lowers productivity, and raises burnout rates among nurses [5].

Burnout presents a severe challenge to the healthcare sector, impacting not only nurses' mental health and job satisfaction but also potentially leading to decreased job performance and high turnover rates. This, in turn, affects the stability of medical teams and the quality of patient care. High levels of burnout increase the incidence of adverse events, reducing patient safety [2, 6, 7]. Patient safety is a critical public health issue, and ensuring safety and quality in cancer care is paramount [8]. With the rising incidence of cancer, nurses in cancer hospitals face immense work pressure. Additionally, when dealing with the grief, fear, and death of cancer patients, nurses may experience compassion fatigue and moral distress, exacerbating burnout [9–11]. Therefore, exploring burnout and its related factors, particularly from a safety perspective, among cancer hospital nurses is of significant importance.

Currently, while the issue of burnout among cancer hospital nurses has received clinical attention, there has been a lack of in-depth analysis from the perspective of nurse-centered safety, focusing on the heterogeneous profiles of burnout and their influencing factors. Latent profile analysis (LPA) is an exploratory, person-centered research method that can identify heterogeneous groups of nurse burnout and the differences between these groups. It also employs objective statistical indicators to measure the accuracy and validity of different profiles, ultimately determining the subgroups of burnout [12, 13].

In this study, we aim to reveal the latent profiles of burnout among cancer hospital nurses and explore its antecedents. Additionally, we seek to investigate the relationships between burnout, nurse safety behaviors, and patient safety competency.

### 1.1. Theoretical Framework and Hypothesis Development

#### 1.1.1. Burnout and Nurse Safety Behaviors

Nurse safety behaviors refer to the conduct exhibited by nursing staff while providing comprehensive care services to patients, including psychological, physiological, and social activities. This encompasses adherence to nursing guidelines and core institutional protocols, as well as the implementation of safety measures [14]. Nurse safety behaviors enhance the quality of care and ensure patient safety. According to Bandura's Social Cognitive Theory (SCT), individual, behavioral, and environmental factors interact and mutually influence one another [15]. Nurses' burnout is an emotional response exhibited by individuals, while safety behaviors are the behavioral state adopted by nurses under the influence of personal and environmental factors. The literature indicates that burnout may reduce nurses' self-efficacy regarding safety behaviors, thereby decreasing its actual execution. In such situations, nurses may neglect their work tasks and overlook critical safety procedures, posing potential threats to patient safety [16, 17]. Furthermore, some studies have found that burnout affects nurses' expectations of work outcomes, reducing their motivation to engage in safety behaviors [18].

#### 1.1.2. Burnout and Patient Safety Competency

Patient safety competency refers to the ability of healthcare professionals to minimize unnecessary harm related to medical care to an acceptable minimum level during the risk control process. It encompasses six aspects: patient-centered care, clinical practice, continuous quality improvement, evidence-based practice, patient safety culture, and safety risk management [19]. Studies showed that patient safety competency can improve the quality of care, reduce the occurrence of nursing errors, and effectively ensure patient safety [20]. Further research indicates that burnout affects nurses' competency in patient safety, potentially leading to decreased cognitive abilities and reduced sensitivity to patient conditions, thereby impacting their ability to respond in complex situations [21]. Emotional exhaustion and work fatigue may impair nurses' ability to recognize and respond quickly to patient conditions, consequently reducing their performance in patient safety [22].

#### 1.1.3. Mediating Role of Patient Safety Competency

From the perspective of SCT, we hypothesized that nurses' patient safety competency may mediate the relationship between burnout and nurse safety behaviors. Specifically, burnout may weaken nurses' self-efficacy and alter their expectations of safety behavior outcomes, which can further affect their patient safety competency [23]. A decline in patient safety competency directly impacts nurses' performance at work, indirectly affecting their patient safety performance by weakening their ability to recognize and handle nurse safety behaviors [24]. Therefore, we hypothesized that nurses' patient safety competency mediates the relationship between burnout and nurse safety behaviors.

  H1: Nurses' patient safety competency mediates the relationship between burnout and nurse safety behaviors.

In summary, this study aims to apply LPA to explore the heterogeneous latent profile characteristics and predictors of burnout among cancer hospital nurses. From the perspective of nursing safety, we aim to use SCT to explore the mediating role of nurses' patient safety competency in the relationship between latent profiles of burnout and nurse safety behaviors.

## 2. Methods

### 2.1. Study Design

A cross-sectional design was used.

### 2.2. Participants and Settings

From April to June 2023, 2092 nurses from tertiary cancer hospitals in 12 provinces of China were selected to participate in this research study using the convenience sampling method. The inclusion criteria were as follows: (i) nurses aged ≥ 18 years and (ii) clinical nurses who had obtained the certificate of a registered nurse. The exclusion criteria were as follows: (i) nurses on maternity or sick leave and (ii) nurses visiting for study and clinical practice.

### 2.3. Procedure

Prior to data collection, the researcher contacted the nursing management teams of cancer hospitals across 12 provinces and cities, securing their agreement to conduct the study. The questionnaire was distributed and collected via the online platform Questionnaire Star. The introductory page provided participants with detailed information on the study's purpose, methodology, and instructions for completion. Participants were informed that their participation was voluntary and anonymous, with the right to withdraw at any time without consequences.

To ensure data integrity, each IP address was restricted to a single submission, and the survey was presented in a sequential, page-turning format, requiring approximately 10 to 15 min to complete. Participants were required to complete all questions before submission, minimizing missing data. The researcher collaborated with department heads to distribute the survey links via departmental WeChat groups, setting a 5-day submission deadline. At the end of the data collection period, two researchers reviewed the submissions, excluding invalid responses. Of the 2240 questionnaires distributed, 2149 were returned. After removing 57 invalid responses, the final sample comprised 2092 valid questionnaires, yielding an effective response rate of 93.4%.

### 2.4. Variables and Instruments

General demographic information was designed by reviewing the literature of previous studies and included gender, age, education level, job position, title, employment form, years of service, monthly income, marital status, weekly working hours, the total number of adverse events experienced in a career, and safety training courses.

#### 2.4.1. Burnout

We utilized the Maslach Burnout Inventory–Human Services Survey (MBI-HSS) to assess burnout among nurses in cancer hospitals. Developed by Maslach and Jackson [25], the MBI-HSS has been validated for reliability and accuracy within the Chinese nursing population by scholars such as Feng et al. [26]. This scale comprises 22 items, divided into three dimensions: emotional exhaustion, depersonalization, and personal accomplishment. Each item is rated on a 7-point Likert scale, ranging from “*never*” to “*every day*,” with scores from 0 to 6. Emotional exhaustion, the core component, represents the dimension of individual stress. Depersonalization reflects the interpersonal relationship dimension, while personal accomplishment pertains to the self-evaluation dimension [27]. Cronbach's alpha coefficient for the entire scale was 0.934, and Cronbach's alpha coefficient for each dimension ranged from 0.865 to 0.934.

#### 2.4.2. Patient Safety Competence

The Patient Safety Competency Self-Rating Scale of Nurses (PSC-SSN) was employed to allow nurses to self-assess their safety competency [28]. The PSC-SSN comprises 29 items distributed across four dimensions: knowledge factors (10 items), system factors (eight items), attitude factors (five items), and skills factors (six items). Each item is rated on a 5-point Likert scale, with “1” representing “*very unclear*” and “5” representing “*very clear*,” resulting in total scores ranging from 29 to 145. Higher scores indicate stronger safety competency. In this study, the PSC-SSN demonstrated excellent reliability, with an overall Cronbach's alpha coefficient of 0.970. Cronbach's alpha coefficients for each dimension ranged from 0.812 to 0.934, indicating robust internal consistency.

#### 2.4.3. Nurse Safety Behaviors

The Nurse Safety Behavior Questionnaire (NSBQ) is designed to measure nurses' behaviors that prevent patient harm and promote patient safety at work [29]. This one-dimensional scale consists of 12 items, each rated on a 5-point Likert scale, with “1” indicating “*never*” and “5” indicating “*always*.” Total scores range from 12 to 60, with higher scores indicating better performance in nurse safety behaviors. In this study, the NSBQ demonstrated excellent reliability, with Cronbach's alpha coefficient of 0.910.

### 2.5. Statistical Analysis

The double data entry was conducted using Epidata 3.1 software, while data analysis was performed using Mplus 8.3 and SPSS 24.0. The MBI-HSS utilized a 7-point Likert scale, and LPA was conducted on the mean scores of its three dimensions. The LPA was executed with Mplus software, employing model fit indices such as the Akaike Information Criterion (AIC), Bayesian Information Criterion (BIC), and sample-size adjusted BIC (aBIC) to assess the profiles' accuracy. Lower values of AIC, BIC, and aBIC indicate superior model fit [30]. Entropy values closer to one suggest more precise classification, with values of 0.80 or higher signifying a classification accuracy of 90% or more [31]. The Lo-Mendell–Rubin adjusted likelihood ratio test (LMRT) and bootstrapped likelihood ratio test (BLRT) were utilized to compare models with different class numbers. These tests assess M-class models against M + 1-class models, where a significant *p* value denotes a better fit for the M + 1 class model [32]. A thorough evaluation of all indices is essential to determine the most optimal model [33].

We used the R3STEP approach to investigate the antecedents of latent burnout profiles. We employed the R3STEP function in Mplus [34, 35], which performs a sequence of multinomial logistic regressions. This approach evaluates whether an increase in covariates would lead to a higher probability of an individual belonging to one class over another. The bootstrap method was employed to assess the mediating effect of nurses' patient safety competency on the relationship between burnout profiles and safety behaviors. Bootstrap samples were drawn 5000 times from the original dataset, and 95% confidence intervals were calculated. If the 95% confidence interval for the standardized path coefficient does not include 0, the mediating effect is deemed significant.

### 2.6. Ethical Consideration

According to Chinese national ethical regulations and institutional guidelines, this study did not require prior ethical review. Specifically, the Measures for the Ethical Review of Life Sciences and Medical Research Involving Humans (2023), issued by the Chinese government, states that noninterventional studies that do not involve medical interventions, biological samples, or personally identifiable sensitive information are exempted from prior ethical review (Article 35). This study strictly adhered to the institutional ethical standards. Participants were informed of the study's purpose and data usage at the time of participation, and written informed consent was obtained. Participation was entirely voluntary. Additionally, this study was conducted anonymously, did not involve any unethical behavior or clinical interventions, and posed no physical or psychological risks to the participants.

## 3. Results

### 3.1. Demographic Information on Survey Respondents

A total of 2092 nurses from cancer hospitals participated in the study, as detailed in Table 1. The majority of participants were female (95.0%), with an average age of 31.89 years (*M* = 31.89, SD = 7.73), and 60.4% were married. Of the respondents, 69.8% held a bachelor's degree in nursing, and 41.2% had between 4 and 9 years of work experience. Most were employed on a contractual basis (73.4%) and earned a monthly income between 6000 and 9000 RMB (57.8%). Additionally, 87.0% were staff nurses, and 71.1% held the title of senior nurse. The majority worked less than 40 h per week (76.7%), had not experienced adverse events in their careers (55.9%), and had received safety training (94.5%).

### 3.2. Model Selection

To analyze burnout among the 2092 nurses from cancer hospitals, we sequentially established latent profile models ranging from 1 to 5 profiles. Based on a thorough evaluation of the latent profile indicators, the three-profile model was identified as the optimal solution, as shown in Table 2. The justification for this selection is as follows: (1) The AIC, BIC, and aBIC values decreased as the number of profiles increased and began to plateau at the two-profile model, (2) an entropy value greater than 0.80 indicated a classification accuracy of 90% for this model, and (3) the LMR and BLRT values were statistically significant (*p* < 0.05). Considering all fit indices and the clinical practical significance, the three-profile model was deemed the most appropriate.

To verify the reliability of the above LPA results, we calculated the average membership probabilities for each profile within the three-profile model. The results showed that the average membership probability for Class 1 was 0.979, for Class 2 was 0.942, and for Class 3 was 0.928, indicating that the model fit with three profiles is reliable [36].

### 3.3. Characteristics and Naming of Latent Profiles of Burnout

**Figure 1:**
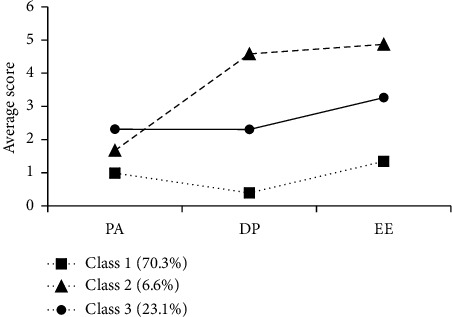
Graphical representation of latent profiles of nurse burnout. Note: EE = emotional exhaustion; DP = depersonalization; PA = personal performance; Class 1 = high achievement stable type; Class 2 = high-efficiency contradictory type; Class 3 = high-pressure adaptive type.

As shown in Figure 1, the three latent profiles of cancer hospital nurses exhibit high response probabilities in the three dimensions of burnout. The profiles were named based on their characteristics. Class 1: Named “high achievement stable type,” this profile includes 1475 nurses (70.3%), the largest proportion. Nurses in this group score low on emotional exhaustion and depersonalization, and high on personal accomplishment. This indicates that they experience less emotional stress and detachment at work while feeling satisfied with their accomplishments. These nurses exhibit the lowest levels of burnout and the highest sense of achievement.

Class 2: Named “high-efficiency contradictory type,” this profile comprises 137 nurses (6.6%). These nurses score the highest on emotional exhaustion and depersonalization but have moderate scores on personal accomplishment. This indicates that they experience high levels of emotional stress and detachment at work, but their sense of accomplishment is not particularly low. These nurses face internal contradictions, despite experiencing significant emotional stress and detachment, they maintain high work efficiency and a moderate level of achievement.

Class 3: Named “high-pressure adaptive type,” this profile includes 480 nurses (23.1%). These nurses score the lowest on personal accomplishment, indicating the highest sense of accomplishment deficit, while scoring moderately on emotional exhaustion and depersonalization. This suggests that they experience a significant lack of accomplishment and moderate levels of emotional stress and detachment at work. These nurses have a poorer ability to adapt to high-pressure environments, exhibiting moderate levels of burnout and the lowest sense of achievement.

### 3.4. Analysis of Antecedents of Latent Profiles of Burnout

Using the R3STEP method, multinomial logistic regression was conducted to examine the relationships between various factors and the latent profiles of occupational burnout. As shown in Table 3, male nurses were more likely to fall into the “high-efficiency contradictory type” and “high-pressure adaptive type” categories compared to the “high achievement stable type.” Nurses in the “high-efficiency contradictory type” typically held lower positions, had lower incomes, worked more than 40 h per week, experienced adverse events, and did not receive safety training. Nurses in the “high-pressure adaptive type” generally held higher professional titles but had lower incomes, worked more than 40 h per week, experienced adverse events, and did not receive safety training.

### 3.5. Mediation Effect Analysis

Before testing for mediation effects, we verified key assumptions to ensure the robustness of the analysis. The Durbin–Watson statistic was approximately two, indicating no autocorrelation among residuals. The variance inflation factor (VIF) values were all below 10, confirming the absence of multicollinearity. To control for potential confounders, we adjusted for gender, age, education level, job position, professional title, employment form, years of service, monthly income, marital status, weekly working hours, the total number of adverse events experienced during a career, and participation in safety training courses. In the mediation effect analysis, the latent profiles of burnout were encoded as dummy variables, while patient safety competency (the mediating variable) and safety behaviors (the dependent variable) were treated as continuous variables. The model demonstrated a good fit, with indices as follows: CFI = 0.957, TLI = 0.895, RMSEA = 0.048, and SRMR = 0.043.

**Figure 2:**
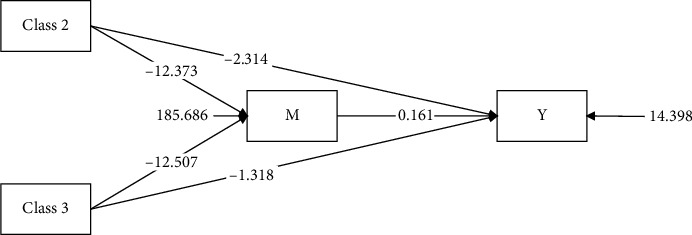
Diagrammatic representation of the mediating effect. Note: Class 2 = high-efficiency contradictory type; Class 3 = high-pressure adaptive type; M = patient safety competency; Y = nurse safety behaviors.

As shown in Table 4 and Figure 2, the results of the mediation analysis indicate that in Path 1, when “high achievement stable type” is used as the reference group, the mediation effect of the “high-efficiency contradictory type” on safety behaviors through patient safety competency is −1.992. The 95% bootstrap confidence interval for this effect is [−2.613, −1.458], which does not include “0,” suggesting a significant partial mediation effect, accounting for 46.3% of the total effect. Furthermore, after incorporating patient safety competency as a mediating variable, the direct effect of the “high-efficiency contradictory type” on safety behaviors is −2.314, with a 95% bootstrap confidence interval of [−3.348, −1.330], again not including “0,” indicating a significant direct effect.

In Path 2, the mediation effect of the “high-pressure adaptive type” on safety behaviors through patient safety competency is −2.013, with a 95% bootstrap confidence interval of [−2.457, −1.634], which does not include “0,” indicating a significant partial mediation effect, accounting for 60.4% of the total effect. This mediation effect is larger than that observed in Path 1. Additionally, after incorporating patient safety competency as a mediating variable, the direct effect of the “high-pressure adaptive type” on safety behaviors is −1.318, with a 95% bootstrap confidence interval of [−1.852, −0.837], again not including “0,” suggesting a significant direct effect.

## 4. Discussion

This multicenter study investigates the latent profiles of burnout among nurses in Chinese cancer hospitals. Our findings identified three distinct latent profile characteristics of burnout: “high achievement stable type,” “high-efficiency contradictory type,” and “high-pressure adaptive type.” We observed variations in the antecedents of these burnout profiles among the nurses. Furthermore, the study confirmed our initial hypothesis that patient safety competency mediates the relationship between latent burnout profiles and safety behavior.

### 4.1. Person-Centered Analysis

#### 4.1.1. Latent Profile Characteristics of Burnout

This study identified three burnout profiles among cancer hospital nurses, providing a nuanced understanding of their occupational stress and highlighting the unique emotional and physical challenges of long-term cancer care. A meta-analysis over the past decade found that burnout among nurses is moderate to high, with a global prevalence of 30%, and this rate is expected to rise [37]. Studies also show a negative association between nurse burnout and patient safety [38]. Factors such as poor working conditions, insufficient time for nursing activities, and excessive workloads contribute to burnout, particularly the emotional exhaustion and depersonalization dimensions, which are most closely linked to patient safety. This relationship has persisted over time, with increasing correlations to the quality of care over the last 30 years [39]. Burnout not only affects nurses' physical and mental health but also compromises care quality, increases medication errors, and threatens patient safety [40].

“High achievement stable type” nurses exhibited low levels of emotional exhaustion and depersonalization, alongside relatively high personal accomplishment. This suggests that they can maintain low emotional stress while deriving a sense of achievement from their work. These nurses likely benefit from strong personal resources and adequate professional support, demonstrating high psychological resilience [41]. Importantly, their resilience and engagement may contribute to consistent patient safety behaviors, as they are more likely to approach tasks with focus and motivation.

“High-efficiency contradictory type” nurses exhibited higher levels of depersonalization, suggesting that while they effectively regulate their emotions and adopt positive coping strategies, they may also detach from their work environment and the recipients of their services. This finding aligns with research on European oncologists, highlighting the significant role of depersonalization in burnout [42, 43]. Cancer nurses face ongoing exposure to the sadness and fear of their patients, as well as negative events such as death, which can lead them to adopt avoidance and relational distancing strategies to manage compassion fatigue and moral distress [9, 10, 17, 44]. Such detachment, while protective against emotional overload, may undermine patient safety by reducing attentiveness, empathy, and engagement in care tasks, potentially leading to errors or missed opportunities for intervention.

“High-pressure adaptive type” nurses reported significantly higher levels of emotional exhaustion and lower scores for personal accomplishment. This indicates that they experience considerable emotional stress in their work. Potential contributing factors include understaffing, insufficient organizational support, heavy workloads, frequent exposure to adverse events, and the generally low cure rates in oncology care. These stressors may trigger negative emotions such as depression and distress, further exacerbating self-doubt and feelings of professional inefficacy [45, 46]. As emotional exhaustion intensifies, these nurses may struggle to maintain the cognitive and emotional resources necessary for delivering safe and effective patient care, increasing the risk of errors and declining quality of care.

These profiles show that burnout is multifaceted, requiring tailored interventions to address its behavioral implications for patient safety competence. For “high achievement stable type” nurses, reinforcing existing support can sustain their positive behaviors and high engagement. For “high-efficiency contradictory type” nurses, psychological support and emotional management interventions can reduce detachment and enhance their connection with patients. For “high-pressure adaptive type” nurses, reducing workload and strengthening support systems can mitigate emotional exhaustion and improve their capacity to focus on patient safety. By addressing the specific characteristics of each burnout profile, these targeted interventions can alleviate burnout, enhance nurse performance, and ultimately improve patient care quality.

#### 4.1.2. Antecedent Factors of Latent Profiles of Burnout

The results of this study indicate that, compared to the “high achievement stable type” reference group, male nurses are more likely to be classified into the “high-efficiency contradictory type” and “high-pressure adaptive type.” Nurses categorized as “high efficiency contradictory type” typically hold lower positions and incomes, work more than 40 h per week, have experienced adverse events, and have not received safety training. Similarly, nurses classified as “high-pressure adaptive type” typically hold higher professional titles but lower incomes and exhibit similar patterns of work hours and experiences.

These findings are consistent with the previous research, highlighting the significant impact of work environment and career development on burnout [47]. Long hours and frequent shifts are known to exacerbate work-family conflicts, increasing burnout risk [48–50]. In China, nurses with higher professional titles often shoulder heavier responsibilities, such as critical care, emergency management, and quality control, leading to significant mental and physical strain [51]. Moreover, frequent exposure to adverse events, insufficient safety training, and lack of organizational support further aggravate burnout levels.

Cancer hospital nurses, in particular, face additional risks such as radiation and exposure to antitumor drugs, which intensify psychological stress and negative emotions [52, 53]. Income disparities, especially in remote regions of China, exacerbate effort-reward imbalances and contribute to burnout. Evidence suggests that implementing high-performance work systems can mitigate nurses' anxiety and depression, enhance job satisfaction, and reduce burnout [54].

To address these challenges, health authorities should allocate human resources more effectively and increase income levels in remote areas. Clinical managers should optimize work schedules, balance workloads with adequate rest, establish safe working environments, and minimize exposure risks. These measures are essential to reduce burnout and promote nurse well-being.

### 4.2. Variable-Centered Analysis

Based on the SCT, this study, with the “high achievement stability type” of nurse burnout as a reference, indicates that patient safety competency partial mediates the effect of nurse burnout latent profiles on their safety behaviors. This underscores the crucial role of enhancing nurses' patient safety competency as a strategy to address nurse burnout.

Nurse safety behaviors and patient safety competency are critical aspects of ensuring safety. Previous studies have showed that burnout can reduce nurse safety behaviors, increase the incidence of adverse events, and lower the quality of care, posing a threat to patient safety [16, 55]. Furthermore, nurses' patient safety competency, including their knowledge, skills, and attitudes toward safety practices, is an essential factor in ensuring patient safety. The research has indicated that systems thinking, psychological safety, teamwork, and professionalism can enhance patient safety competency [56, 57]. The negative impact of nurse burnout on patient safety competency implies that enhancing nurses' patient safety competency can directly improve their safety behaviors and play a crucial role in addressing nurse burnout. For example, through systematic safety training and professional development support, nurses' self-efficacy and professional skills can be enhanced, thereby reducing the impact of burnout.

Healthcare institutions should address nurses' burnout issues by indirectly improving their safety behaviors by enhancing their patient safety competency. This not only helps reduce the incidence of burnout but also enhances overall nursing quality and patient safety levels.

## 5. Conclusion

In summary, the results of this study not only reveal the various manifestations and causes of nurse burnout but also provide theoretical support through variable-centered analysis, indicating the significant role of patient safety competency in the relationship between burnout latent profiles and nurse safety behaviors. Nursing managers should adopt personalized interventions for different types of burnout, focusing on enhancing nurses' patient safety competency to improve their burnout status and increase nurse safety behaviors.

### 5.1. Implications for Nursing Management

Nursing managers should develop targeted interventions based on the three identified burnout profiles (“high achievement stable type,” “high-efficiency contradictory type,” and “high-pressure adaptive type”) and provide personalized support to address specific needs and vulnerabilities. This study highlights the crucial role of patient safety competency in mediating the relationship between burnout and safety behaviors, emphasizing the importance of comprehensive safety training programs. Nursing managers should prioritize ongoing education and training initiatives, optimize work schedules to prevent burnout, and ensure adequate staffing levels. Additionally, providing extra support for high-risk groups, fostering a positive work environment, and promoting work-life balance are essential strategies.

The significance of this research lies in its integration of person-centered and variable-centered approaches, offering new insights into the multifaceted nature of burnout and its impact on nurse safety behaviors. By implementing these measures, nursing management can significantly enhance nurse well-being and job satisfaction, ultimately improving patient care quality in cancer hospitals.

### 5.2. Limitations

Although the findings of this study offer practical insights into cancer nurses' burnout, several limitations should be noted. First, data were based on self-reported questionnaires, which may be prone to social desirability and recall biases. Future studies could incorporate objective data (e.g., job performance assessments) to validate the results. Second, the cross-sectional design prevents the determination of causal relationships. Longitudinal designs are needed to better understand the dynamics between burnout, patient safety competency, and safety behaviors. Additionally, the sample was limited to a specific region, which affects the external validity of the findings. Expanding the sample in future research would improve generalizability. Finally, path analysis models assume reliable and valid measurements, but measurement errors may impact the model's accuracy. Future studies should address measurement error in latent variable modeling or use multiple measures to reduce its impact, thereby enhancing the model's reliability.

### 5.3. Future Directions

Future research should further explore the following aspects. Firstly, an in-depth investigation of the formation mechanisms of different types of burnout and their long-term impacts on nurses' professional development to develop more effective intervention strategies. Secondly, exploration of specific measures to enhance nurses' patient safety competency, such as safety training and psychological support, to mitigate the negative effects of burnout. Lastly, it is recommended to adopt longitudinal research designs to dynamically track changes in nurse burnout, patient safety competency, and safety behaviors to further validate their causal relationships.

## Figures and Tables

**Table 1:** Demographic information on survey respondents (mean SD or *n* [%]).

Variable	Number	Class 1	Class 2	Class 3
		*n* = 1475	*n* = 137	*n* = 480
Age (year)	31.89 ± 7.73	32.06 ± 8.09	30.66 ± 7.70	31.75 ± 6.50

*Gender*				
Male	104 (5.0)	51 (3.5)	18 (13.1)	35 (7.3)
Female	1988 (95.0)	1424 (96.5)	119 (86.9)	445 (92.7)

*Education level*				
Junior college	357 (17.1)	273 (18.5)	16 (11.7)	68 (14.2)
Faulty-to-undergraduate	246 (11.8)	175 (11.9)	17 (12.4)	54 (11.3)
Undergraduate	1461 (69.8)	1010 (68.5)	102 (74.5)	349 (72.7)
≥ Postgraduate	28 (1.3)	17 (1.2)	2 (1.5)	9 (1.9)

*Job position*				
None	1821 (87.0)	1292 (87.6)	120 (87.6)	409 (85.2)
Tutor	164 (7.8)	102 (6.9)	16 (11.7)	46 (9.6)
Head nurse	107 (5.1)	81 (5.5)	1 (0.7)	25 (5.2)

*Title*				
Nurse	536 (25.6)	426 (28.9)	26 (19.0)	84 (17.5)
Nurse practitioner	833 (39.8)	547 (37.1)	71 (51.8)	215 (44.8)
Senior nurse or head nurse and above	723 (34.6)	502 (34.0)	40 (29.2)	181 (37.7)

*Years of service*				
< 4	503 (24.0)	388 (26.3)	31 (22.6)	84 (17.5)
4∼	862 (41.2)	546 (37.0)	72 (52.6)	244 (50.8)
10∼	500 (23.9)	350 (23.7)	31 (22.6)	119 (24.8)
20∼	227 (10.9)	191 (12.9)	3 (2.2)	33 (6.9)

*Employment form*				
Formal incorporation	350 (16.7)	272 (18.4)	14 (10.2)	64 (13.3)
Contract employment	1536 (73.4)	1050 (71.2)	111 (81.0)	375 (78.1)
Personnel agency	206 (9.8)	153 (10.4)	12 (8.8)	41 (8.5)

*Monthly income (RMB)*				
1000∼	320 (15.3)	214 (14.5)	30 (21.9)	76 (15.8)
6000∼	1209 (57.8)	806 (54.6)	89 (65.0)	314 (65.4)
10,000∼	563 (26.9)	455 (30.8)	18 (13.1)	90 (18.8)

*Marital status*				
Married	1263 (60.4)	882 (59.8)	80 (58.4)	301 (62.7)
Single	829 (39.6)	593 (40.2)	57 (41.6)	179 (37.3)

*Weekly working hours*				
≤ 40	1604 (76.7)	1224 (83.0)	74 (54.0)	306 (63.8)
> 40	488 (23.3)	251 (17.0)	63 (46.0)	174 (36.3)

*Adverse incident experience*				
None	1169 (55.9)	921 (62.4)	51 (37.2)	197 (41.0)
Yes	923 (44.1)	554 (37.6)	86 (62.8)	283 (59.0)

*Safety training courses*				
Attended	1976 (94.5)	1412 (95.7)	122 (89.1)	442 (92.1)
Not-attended	116 (5.5)	63 (4.3)	15 (10.9)	38 (7.9)

*Note:* Class 1 = high achievement Stable Type; Class 2 = high-efficiency contradictory type; Class 3 = high-pressure adaptive type.

**Table 2:** Burnout latent profile model fit metrics.

Model	LL	AIC	BIC	aBIC	Entropy	LMR *p* value	BLRT *p* value	Class (%)
Class 1	−10741.637	21495.274	21529.150	21510.087	NA	NA	NA	1
Class 2	−9633.623	19287.247	19343.706	19311.935	0.914	< 0.0001	< 0.0001	76.3/23.7
Class 3	−9204.753	18437.505	18516.548	18472.068	0.922	< 0.0001	< 0.0001	70.3/6.6/23.1
Class 4	−8948.567	17933.133	18034.759	17977.571	0.927	0.0008	< 0.0001	23.9/6.6/66.1/3.4
Class 5	−8637.085	17318.170	17442.379	17372.483	0.871	0.1079	< 0.0001	3.0/46.9/28.1/17.6/4.4

*Note:* aBIC = sample-size-adjusted BIC.

Abbreviations: AIC = Akaike Information Criteria; BIC = Bayesian Information Criteria; BLRT = bootstrapped likelihood ratio tests; LL = log-likelihood; LMR = LoMendell–Rubin.

**Table 3:** Adjusted logit differences from multinomial logistic regression analysis (R3STEP).

Profile comparison		Class 2 vs. Class 1	Class 3 vs. Class 1	Class 3 vs. Class 2
Age (year)		0.015	0.005	−0.011

Gender	Male (ref.)			
	Female	−1.140⁣^∗^	−0.624⁣^∗^	0.517

Education level	Junior college (ref.)			
	Faulty-to-undergraduate	0.238	−0.171	−0.409
	Undergraduate	0.077	−0.180	−0.257
	≥ Postgraduate	0.835	0.376	−0.459

Job position	None (ref.)			
	Tutor	0.396	0.187	−0.209
	Head nurse	−17.430⁣^∗∗^	−0.043	17.387

Title	Nurse (ref.)			
	Nurse practitioner	0.562	0.469	−0.093
	Senior nurse or head nurse and above	0.393	0.643⁣^∗^	0.249

Years of service	< 4 (ref.)			
	4∼	−0.162	0.333	0.495
	10∼	−0.379	0.068	0.446
	20∼	−2.728	−0.489	2.240

Employment form	Formal incorporation (ref.)			
	Contract employment	−0.635	0.043	0.678
	Personnel agency	−0.523	0.031	0.554

Monthly income (RMB)	1000 (ref.)			
	6000	−0.450	−0.233	0.218
	10,000	−0.976⁣^∗^	−0.563⁣^∗^	0.413

Marital status	Married (ref.)			
	Single	0.202	0.234	0.033

Weekly working hours	≤ 40 (ref.)			
	> 40	1.132⁣^∗∗^	0.838⁣^∗∗^	−0.294

Adverse incident experience	None (ref.)			
	Yes	0.948⁣^∗∗^	0.676⁣^∗∗^	−0.272

Safety training courses	Attended (ref.)			
	Not-attended	0.982⁣^∗^	0.665⁣^∗^	−0.317

*Note:* The values represent adjusted logit differences between latent profiles (e.g., Class 2 vs. Class 1) derived using the R3STEP method, which corrects for classification errors in latent profile analysis. Positive values indicate that the logit value for the comparison group (e.g., Class 2) is higher than the reference group (e.g., Class 1), while negative values indicate the opposite. Class 1 = high achievement stable type; Class 2 = high-efficiency contradictory type; Class 3 = high-pressure adaptive type.

⁣^∗^*p* < 0.05.

⁣^∗∗^*p* < 0.001.

**Table 4:** Analysis of mediating effects of patient safety competency (unstandardized).

Class 1 (ref.)	Impact	95% CI	
		LLCI	ULCI
Class 2 ⟶ M ⟶ Y	−1.992^a^	−2.613	−1.458
Class 2 ⟶ Y	−2.314^a^	−3.348	−1.330
Class 3 ⟶ M ⟶ Y	−2.013^a^	−2.457	−1.634
Class 3 ⟶ Y	−1.318^a^	−1.852	−0.837

*Note:* Class 1 = high achievement stable type; Class 2 = high-efficiency contradictory type; Class 3 = high-pressure adaptive type; M = patient safety competency; Y = nurse safety behaviors.

^a^Significant mediation effect.

## Data Availability

The data that support the findings of this study are available on request from the corresponding author. The data are not publicly available due to privacy or ethical restrictions.
